# The Shape of Posterior Sclera as a Biometric Signature in Open-angle Glaucoma: An Intereye Comparison Study

**DOI:** 10.1097/IJG.0000000000001573

**Published:** 2020-06-16

**Authors:** Yong Chan Kim, Yong Ho Koo, Hyung Bin Hwang, Kui Dong Kang

**Affiliations:** * Department of Ophthalmology, College of Medicine, The Catholic University of Korea, Incheon St. Mary’s Hospital, Incheon; † Department of Ophthalmology, College of medicine, Chuncheon Sacred Heart Hospital, Hallym University, Chuncheon-si, Gangwon-do, Republic of Korea

**Keywords:** glaucoma, posterior sclera, intereye comparison

## Abstract

**Purpose:**

To characterize intereye differences in posterior segment parameters and determine their significance in open-angle glaucoma patients with unilateral damage.

**Methods:**

Both eyes from 65 subjects without any nerve damage and 43 patients undergoing treatment for unilateral open-angle glaucoma were included in this study. A 12.0×9.0×2.6 mm volume of the posterior segment in each eye was scanned with swept-source optical coherence tomography. Coronally reconstructed optical coherence tomography images were analyzed to determine the deepest point of the eye (DPE), which we then calculated the distance (Disc-DPE distance), depth (Disc-DPE depth), angle (Disc-DPE angle) from the optic disc center to the DPE. Posterior pole shape was analyzed measuring the posterior pole-cross-sectional area, posterior pole-horizontal width (PP-HW), and posterior pole-vertical width) of the posterior pole. These measurements and their intereye absolute difference (IAD; absolute difference in measurements between the right and left eyes) values were compared between the healthy and unilateral glaucomatous patients.

**Results:**

The posterior sclera measurements, including the Disc-DPE distance, Disc-DPE depth, and posterior pole-cross-sectional area, were significantly different between the unilateral glaucoma eyes and contralateral healthy eyes (*P*=0.043, *P*=0.035, and *P*=0.049, respectively). By contrast, none of the intereye differences in optic nerve head parameters were significant in the unilateral glaucoma patients. In comparison with the IAD values, the baseline intraocular pressure and PP-HW of the posterior segment showed significant differences between the healthy and the unilateral glaucoma patients (*P*=0.019 and *P*=0.036, respectively). A multivariate analysis showed that a larger baseline intraocular pressure IAD [odds ratio (OR), 1.381; *P*=0.009)] and larger PP-HW IAD (OR, 1.324; *P*=0.032) were significantly associated with the presence of glaucoma.

**Conclusions:**

Compared with the fellow healthy eyes, glaucomatous eyes had larger and more steeply curved posterior poles, which represent a structural variation of the posterior sclera that might be associated with glaucomatous optic neuropathy.

## Abstract 2

Supplemental Digital Content is available in the text.

A glaucoma is a group of progressive optic neuropathies that have in common a slow progressive degeneration of retinal ganglion cells and their axons, resulting in a distinct appearance of the optic disc and a concomitant pattern of visual loss.^1^ Although glaucoma frequently occurs with an elevation of intraocular pressure (IOP), a significant number of patients with IOP in the normal range also develops glaucomatous optic nerve damage.^2^ In contrast, a sufficient reduction of IOP in high-tension glaucoma does not ensure a slower progression either.^3^

It has recently been suggested that the central pathophysiology of glaucoma involves IOP-related stress and strain on the load-bearing connective tissues of the peripapillary sclera, scleral canal wall, and lamina cribrosa (LC).^4^ Thus, high IOP over time (ie, during the course of aging) may induce constant remodeling of the load-bearing scleral extracellular matrix, resulting in differences in durability therein among individuals.^5^,^6^ Numerous experimental models and computer-aided engineering simulations have also validated these characteristic microstructural alterations and deformations related to glaucomatous damage.^7^–^10^ On the basis of these reports, our group has characterized the effects in the entire posterior sclera using the same conceptual framework; the studies described above focused only on the optic nerve head (ONH) and its contiguous areas, which constitutes only a part of the load-bearing posterior segment.^11^,^12^ Although the ONH is the most important anatomic area directly supporting the retinal ganglion cell axons, it is also anatomically connected to, and interacts with, the surrounding posterior sclera.^13^,^14^ As in other load-bearing connective tissues in the eye, the sclera is an extracellular matrix constantly being remodeled according to the IOP load over time.^15^,^16^

This study is a follow-up to our research on the posterior sclera biometrics of glaucoma using the novel parameter deepest point of eye (DPE).^12^,^17^ We have suggested that glaucomatous eyes had the DPE situated deeply posterior to the optic disc, which was consistent with the comparison between the glaucomatous and nonglaucomatous eyes, and also with the progressive and nonprogressive glaucomatous eyes.^11^,^18^ Moreover, it is well documented that peripapillary and ONH structures change over time in childhood.^19^ The larger parapapillary atrophy measured in unilateral open-angle glaucoma (OAG) eyes could be the resultant of the larger structural change of the sclera.^20^ Therefore, we have recruited a group of patients with OAG affected in their 1 eye and their fellow eye without any sign of glaucomatous damage, and compared the biometric differences of the ONH and the posterior sclera in both eyes of unilateral OAG to figure out the significance of posterior sclera structure in glaucoma.

## METHODS

### Study Participants

This cross-sectional analysis included patients who visited Incheon Saint Mary’s Hospital Glaucoma Clinic from June 2016 and July 2019. This study was carried out in accordance with the recommendation of the Declaration of Helsinki for biomedical research involving human subjects. The study protocol reviewed and approved by The Catholic University of Korea institutional review board. All the data included in the study were analyzed after deidentification.

All subjects were required to have a best-corrected visual acuity of at least 20/40, spherical refraction of −6.0 to +3.0 diopters (D), and a cylinder correction of −3.0 to +3.0 D. Those with a history of ocular surgery other than uncomplicated cataract surgery, intraocular diseases (eg, diabetic retinopathy, uveitis, retinal vessel occlusion), history or evidence of optic neuropathies other than glaucoma or congenital anomalies of the optic disc, signs of pathologic myopia including myopic choroidal neovascularization, lacquer crack, angioid streaks, extremely myopic eyes with an axial length >30 mm, and evident posterior staphyloma were excluded. The age were matched between the healthy control group and the unilateral glaucoma group. Eyes with poor image quality (under 75 in B-scan mode) in which the DPE was not delineated clearly on optical coherence tomography (OCT) images were excluded.

The subjects included in the unilateral glaucoma group were required to have OAG with corresponding visual field (VF) defect in 1 eye and the other eye with no manifest glaucomatous optic neuropathy (ie, unilateral manifest OAG). The diagnosis of OAG was according to the following criteria: typical glaucomatous optic neuropathy disc changes (such as localized or diffuse rim thinning, disc hemorrhage, and notch in the rim) as seen on stereo disc photography; typical glaucomatous retinal nerve fiber layer (RNFL) changes as seen on either stereo disc photography or red-free RNFL images; corresponding glaucomatous VF loss; open anterior chamber on nonindentation gonioscopy in primary position. A glaucomatous VF was defined as meeting 2 or more of the following criteria: outside normal limits on the glaucoma hemifield test; the presence of at least 3 contiguous test points within the same hemifield on the pattern deviation plot at *P*-value <5% with at least one of these points <1%; a pattern standard deviation (PSD) of probability <5%. These VF defect patterns were confirmed by 2 consecutive reliable tests (fixation loss rate <20%, as well as false-positive and false-negative error rates <15%). The contralateral healthy eye had to have an open angle on gonioscopy, an optic disc appearance without glaucomatous optic neuropathy changes, and a VF that does not meet the glaucomatous VF definition.

All subjects underwent a complete ophthalmic examination at initial visit: visual acuity assessment, refraction, slit-lamp biomicroscopy, gonioscopy, Goldmann applanation tonometry (Haag-Streit), dilated funduscopic examination, standard automated perimetry (Humphrey VF Analyzer; 30–2 Swedish Interactive Threshold Algorithm; Carl Zeiss Meditec Inc., Dublin, CA), central corneal thickness (CCT) by ultrasound pachymetry (Tomey Corporation, Nagoya, Japan), axial length with ocular biometry (IOL Master; Carl Zeiss Meditec Inc.), stereo disc photography and red-free RNFL photography (Canon, Tokyo, Japan), standard automated perimetry (Humphrey Field Analyzer with 24-2 Swedish interactive threshold algorithm; Carl Zeiss Meditec Inc.), and swept-source optical coherence tomography (SSOCT, DRI OCT Triton; Topcon Corporation, Tokyo, Japan). Subjects had regular follow-up every 3 to 6 months with slit-lamp examinations using a 78 D lens or stereo disc photography.

Goldmann applanation tonometry was used to measure IOP at the baseline (before initiation of topical antiglaucoma medication) and at every follow-up visit thereafter. Diurnal measurements of IOP were obtained at 9:00 am and 11:00 am (twice at each visit), and the average of these 2 measurements were defined as the baseline IOP. On diagnosis, patients received topical ocular hypotensive medications on their OAG-affected eyes with the treatment target matched to a 20% reduction from baseline IOP. When this was not accomplished, further treatment decisions were made by the treating physician. Mean follow-up IOP was calculated by averaging all IOP measurements during the follow-up period. IOP fluctuation was defined as the SD of the follow-up IOP value.

### Biometric Evaluation of Posterior Sclera

**FIGURE 1:**
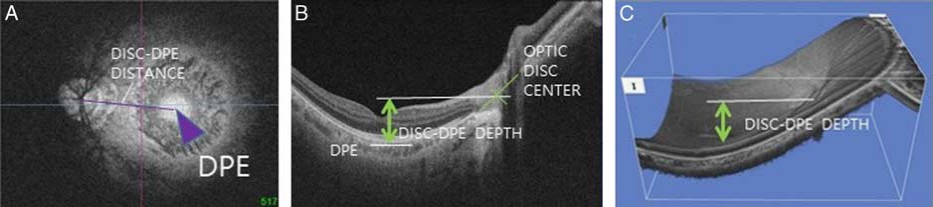
Deepest point of the eye (DPE) and measurement landmarks in the reconstructed coronal scan. Bruch’s membrane (BM) seemed as a hyper-reflective round plane with no hyporeflective vitreous cavity inside BM, and with the nonhomogenous hyporeflective choroid surrounding the outside of the BM. This landmark is designated as the DPE (A). The Disc-DPE distance (A) was quantified as the lineal distance from the optic disc center to the DPE measured along the same en-face image as the DPE. The Disc-DPE depth (B, C) was calculated by counting the number of coronal sections from the interface of the DPE to the interface of the optic disc center.

Unlike other parts of the eye, the posterior sclera is located in the most inner part of the eye covered with orbital bones and fat tissue, which makes it difficult to directly parametrize its structural figure. We have used DPE as a biometric reference point to represent the structure of the posterior sclera. This method is described previously.^18^ In brief, the scanning protocol consisted of 256 B-scans centered on the fovea, providing the image of the posterior segment 12 mm horizontally, 9 mm vertically, and 2.6 mm in depth. The system software reconstructs the 2.6 mm depth of the posterior segment of the eyeball with 1000 consecutive coronal images from the anterior to the posterior without any flattening error. In the reconstructed coronal scan, the Bruch membrane (BM) seems hyper-reflective circle with vitreous cavity inside showing a hyporeflection. The hyper-reflective circle becomes smaller as the coronal view goes into the posterior direction and when the section reaches the most posterior part of the eye the circle becomes a hyper-reflective round plane without hyporeflective vitreous cavity inside of BM and with the inhomogenous hyporeflective choroid surrounding the outside of BM. This landmark is designated as the DPE (Fig. 1). In other words, the DPE was the deepest and the most posterior BM/choroid interface in the anteroposterior axis that showed no vitreous cavity at its center, and also with the least amount of the BM shown inside the choroid tissue (Fig. 1).

DPE position was used to describe the posterior pole, which we define as the part of the posterior segment that is posterior to the optic disc center. We used the optic disc center as another reference point because the SSOCT software automatically indicates the center of the optic disc as a green cross based on the margin of BM. We then used this anatomy to measure the relative distance from the optic disc center, the amount of angular position from the horizontal line crossing the OCT-defined optic disc center, and the amount of anteroposterior depth from the optic disc center. The software also provides a precise point-to-point correlation between the individual reconstructed OCT images with the fundus photographic images. BM has been proposed as the biometric landmark for neuroretinal rim measurements because of its anatomic consistency.^21^,^22^ Using the deepest point of BM is consistent with this supposition. Under these suppositions, the Disc-DPE distance was quantified as the lineal distance from the optic disc center to the DPE measured along the same coronal section as the DPE. The Disc-DPE angle was measured as the angle from the horizontal meridian crossing the OCT-defined optic disc center to the linear line from the OCT-defined optic disc center to the DPE center. Third, the Disc-DPE depth was calculated by counting the number of coronal sections from the interface of the DPE to the interface of the optic disc center and converting the number of sections into micrometers by assuming a depth of 2.6 μm for each coronal section. The 2.6 μm for each coronal section depth was on the assumption that the effective scan depth of 2.6 mm was divided evenly in 1000 coronal sections.

**FIGURE 2:**
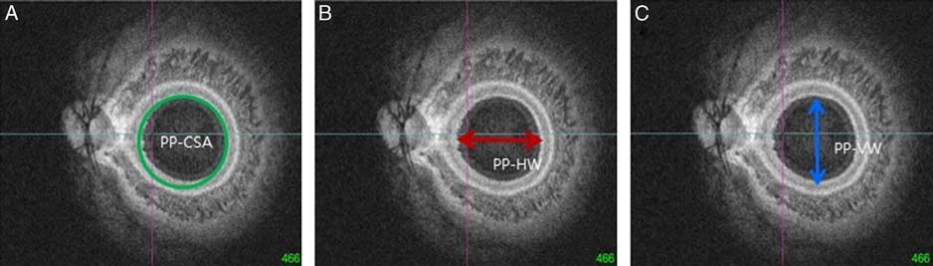
The shape and curvature of the posterior segment was represented using the biometric measurements obtained at the center of the optic disc in the reconstructed coronal section. Posterior pole-cross-sectional area (PP-CSA) was the total cross-sectional area of the vitreous cavity measured at the height of the optic disc (A). The posterior pole-horizontal width (PP-HW) was the horizontal width of the vitreous cavity, measured at the height of the optic disc (B). The posterior pole-vertical width (PP-VW) was the vertical width of the vitreous cavity measured at the height of the optic disc (C).

Moreover, the shape and curvature of the posterior segment were represented with the biometric measurements at the height of the optic disc center in the reconstructed coronal section. The posterior pole-cross-sectional area (PP-CSA) was the total cross-sectional area of the vitreous cavity measured at the height of the optic disc center. The posterior pole-horizontal width (PP-HW) measured the largest horizontal width of the inner posterior sclera cavity using a coronal cut of BM as demarcation. The posterior pole-vertical width (PP-VW) measured the largest vertical width of the inner posterior sclera cavity using a coronal cut of BM as demarcation. Both PP-HW and PP-VW were measured the coronal scan at the height of the optic disc center (Fig. 2). Two observers (Y.C.K. and Y.H.K.) measured the parameters manually using the inbuilt intrinsic caliper function of the DRI OCT Triton software in a blinded manner.

### Biometric Measurement of ONH

The ONH configuration was measured with the degree of optic disc tilt from the horizontal and vertical cross-sectional images, the amount of torsion degree of the optic disc, and the horizontal length of β zone peripapillary atrophy (PPA) and γ zone PPA. The optic disc tilt measurement procedure has been described in detail elsewhere.^23^ In brief, the disc photographs acquired simultaneously by the SSOCT were overlapped to the horizontal scan of the OCT and the 2 glaucoma specialists (Y.C.K. and K.D.K.) marked the nasal and the temporal clinical disc margin points at the fundus photographic images, which automatically marks the equivalent points at the horizontal scan of the OCT. A line connecting the 2 points marking the clinical disc margin on the cross-sectional images was defined as the ONH plane. A line connecting the inner tips of BM on each side of the ONH plane on the cross-sectional images was drawn as the reference plane. The degree of tilt was defined as the angle between the reference plane and the ONH plane. Angle measurements were performed by 2 observers (Y.C.K. and K.D.K.) with the built-in caliper of the software. A positive degree of horizontal tilt indicated a temporal tilt, and a negative value indicated a nasal tilt. The degree of vertical tilt was also measured from a vertical cross-sectional image in the same way as described above. A positive degree of tilt indicated an inferior tilt, and a negative value indicated a superior tilt. Second, the optic disc was defined as torsioned when the axis of the maximum optic disc diameter was not aligned with the vertical meridian, similar to the Blue Mountains study.^24^,^25^ The vertical meridian was considered a vertical line 90 degrees from a line connecting the fovea to the center of the disc. A positive torsion value indicated counterclockwise torsion in the right eye format, and a negative value indicated clockwise torsion in the right eye format. Third, the PPA biometric measurement procedure has been described in detail elsewhere.^26^ In brief, β zone PPA, which was measured between BM endpoint to the beginning of the retinal pigment epithelium with underlying BM at the center of the optic disc. γ zone PPA was measured from the anterior scleral canal opening to BM endpoint. Lastly, the anterior LC depth was determined by measuring the distance from the BM opening plane to the level of the anterior LC surface.^27^ The anterior borders of the LC were defined by the highly reflective structure below the optic cup. The line connecting the 2 termination points of BM edges at the optic disc center was used as a reference plane for LC depth measurements. LC depth was the average of the distance from the reference line to the anterior LC surface, estimated at 3 B-scans (mid-superior, center, mid-inferior). The laminar thickness was defined as the distance between the anterior and posterior borders of the highly reflective region at the vertical center of the ONH in the horizontal OCT cross-sectional B-scans.^28^ Anterior scleral canal opening was measured as the line connecting the 2 termination points of BM edges at the optic disc center. Parameters were measured with the intrinsic caliper at the optic disc center. The disagreements were resolved by consensus between the 2 ophthalmologists or a third adjudicator (H.B.H.).

### Measurement Reproducibility

Although the position of the DPE is a parameter that represents a 3-dimensional coordinates, it is reproducible just like every other ocular biometric parameters if a proper fixation to the OCT apparatus is achieved. The most effective way to ensure that the fixation is achieved in the scan is to verify the reproducibility of the biomarkers.^29^,^30^ Twenty eyes underwent intraclass correlation test on 2 separate scans. The intraclass correlation coefficients were determined by 2-way mixed effect model.^31^ The intraclass correlation scores over 0.75 are considered excellent.^32^ The reproducibility is excellent because the DPE position is accountable whenever the visual axis is fixed at the same target on each scan. In addition, SSOCT has real-time eye tracking that has been known to eliminate eye motion and minimize artifacts by fixating on the fovea on each scan. Thus, like any other structures of the fundus photograph, DPE position is reproducible as long as the proper fixation is achieved.

### Statistical Analysis

Baseline characteristics were reported in mean±SD values. The normally distributed data between the paired eyes of a subject were compared by paired *t* test and the categorical data were analyzed by χ^2^ test. Intereye absolute difference (IAD) was the absolute difference between right and left eye measures. The IAD was compared by the independent *t* test between the healthy and unilateral glaucoma groups. Univariate and multivariate conditional logistic regression analyses were performed to identify factors associated with the presence of glaucoma. Variables with *P*-values <0.1 on univariate analysis were included in the multivariate analysis, and the backward variable selection was utilized to obtain the final multivariable model. The regression analyses were first done with the actual measured value and another with the value of absolute intereye difference. Probability values of *P*<0.05 were considered statistically significant. The statistical analysis was performed using the SPSS statistical package, version 22.0 (IBM Corp., Chicago, IL).

## RESULTS

This study initially involved 74 subjects without any glaucomatous damage in either eye and 49 patients diagnosed with OAG with unilateral glaucomatous damage. Of these, 15 patients were excluded because the image quality of their OCT scans was too poor to allow clear visualization of DPE. Eventually, a total of 130 eyes of 65 healthy controls and 86 eyes of 43 unilateral glaucoma patients were enrolled in the current study. The mean participants’ age was 52.41±16.27 years. The analysis based on 20 independent cases of intervisit reproducibility were conducted twice on different days by 2 authors (see Table, Supplemental Digital Content 1, http://links.lww.com/IJG/A400, which demonstrates reproducibility). Measured parameters had excellent reproducibility.

The demographical characteristics of the study subjects are represented in Table 1. The healthy and the unilateral glaucoma group had no significant differences in their demographic composition of age at diagnosis, female gender, diabetes mellitus, hypertension, family history of glaucoma and cold extremities, or migraine. In their biometric comparison between the paired eyes of the same individual, healthy controls had no significant differences in their axial length, CCT, baseline IOP, mean follow-up IOP, IOP fluctuation, IOP reduction from baseline, RNFL thickness globally and in each sector, automated perimetry mean deviation value and PSD value. In contrast, patients with unilateral glaucoma damage had significant intereye biometric differences with thinner CCT (*P*=0.006), higher baseline IOP (*P*=0.048), thinner RNFL thickness in every sector (global, *P*<0.006; superior, *P*<0.001; nasal, *P*=0.001; inferior, *P*<0.001; temporal, *P*=0.002), and worse MD and PSD value (both *P*<0.001, respectively).

**TABLE 1:** Demographic and General Ocular Biometric Characteristics of the Study Subjects

	Healthy Controls (n=65)			Unilateral Glaucoma (n=43)			
Variables	Right Eye	Left Eye	P*	Healthy Eye	Glaucoma Eye	P*	*P*†
Age at diagnosis (y)	52.30±17.14			52.53±15.08			0.579
Female gender (%)	38 (58.46)			19 (44.18)			0.458
Diabetes mellitus (%)	16 (24.61)			9 (20.93)			0.660
Hypertension (%)	22 (33.84)			17 (39.53)			0.551
Family history of glaucoma (%)	3 (4.61)			7 (16.27)			0.057
Cold extremities or migraine (%)	7 (10.76)			11 (25.58)			0.127
Disc hemorrhage (%)	0 (0)	0 (0)		0 (0)	2 (4.65)	0.224	
Axial length (mm)	24.57±1.62	24.73±1.66	0.316	24.27±1.28	24.32±1.40	0.426	
Refractive error (D)	−1.49±2.86	−1.61±3.02	0.463	−1.93±2.52	−2.13±2.35	0.315	
Central corneal thickness (μm)	544.31±44.48	541.61±42.26	0.059	524.97±35.07	519.87±35.61	**0.006**	
Baseline IOP (mm Hg)	16.52±3.64	16.73±3.54	0.366	16.25±2.90	18.16±8.05	**0.048**	
Mean follow-up IOP (mm Hg)	16.19±3.30	16.39±3.46	0.319	15.30±2.64	15.48±3.73	0.676	
IOP fluctuation (mm Hg)	0.90±1.13	0.73±0.81	0.119	1.62±0.98	1.68±0.97	0.692	
IOP reduction from baseline (%)	1.04±11.22	1.54±10.19	0.737	2.91±13.04	9.72±19.38	**0.031**	
RNFL thickness (μm)							
Global	102.51±13.21	101.10±13.48	0.358	96.37±12.01	74.55±14.58	**<0.001**	
Superior sector	122.52±23.39	126.10±20.56	0.185	119.18±17.52	89.06±25.64	**<0.001**	
Nasal sector	76.07±16.58	70.87±17.65	0.070	70.44±14.77	61.58±19.44	**0.001**	
Inferior sector	129.96±19.11	124.03±23.78	0.743	118.88±18.03	77.97±24.64	**<0.001**	
Temporal sector	83.83±13.82	83.64±13.79	0.910	77.16±12.86	69.55±14.46	**0.002**	
Automated perimetry (dB)							
MD	−0.48±1.21	−0.62±1.30	0.434	−0.79±1.82	−5.38±4.25	**<0.001**	
PSD	1.86±0.66	1.94±0.87	0.845	1.86±0.66	6.13±3.86	**<0.001**	

Data are presented as mean±SD unless otherwise indicated.

*Paired *t* test for continuous variables.

†χ^2^ test for categorical variables.

Statistically significant values (*P*<0.05) are shown in bold.

dB indicates decibel; IOP, intraocular pressure; MD, mean deviation of perimetry; PSD, pattern standard deviation of perimetry; RNFL, retinal nerve fiber layer.

Measurements of the ONH and posterior pole between both eyes of the healthy controls and between glaucomatous eyes and fellow healthy eyes were compared (Table 2). Although healthy controls did not have significant intereye differences in both ONH and posterior sclera biometric measures, the unilateral glaucoma patients had significant intereye differences in their posterior pole biometric measurements. Compared with their contralateral healthy eyes, mean Disc-DPE distance were significantly longer (2.12±0.96 vs. 2.54±1.57, *P*=0.043), Disc-DPE depth were significantly deeper (67.28±51.35 vs. 110.26±141.85, *P*=0.035), PP-CSA area were significantly larger (4.09±5.46 vs. 6.99±1.11, *P*=0.049).

**TABLE 2:** Comparison of the Posterior Segment Biometrics Between Both Eyes of the Healthy Controls and Between Glaucomatous Eyes and Fellow Healthy Eyes

	Healthy Controls (n=65)			Unilateral Glaucoma (n=43)		
Variables	Right Eye	Left Eye	P*	Healthy Eye	Glaucoma Eye	P*
ONH characteristics						
Horizontal tilt (deg.)	13.75±8.10	12.00±9.00	0.144	15.55±7.25	17.07±6.60	0.248
Vertical tilt (deg.)	9.04±8.31	8.58±8.63	0.675	12.09±8.03	13.78±9.37	0.348
Disc torsion (deg.)	−3.78±8.25	−2.26±11.92	0.370	−1.12±16.47	−0.05±17.85	0.627
β zone PPA (μm)	178.93±152.40	179.69±150.91	0.969	151.72±139.81	178.37±144.47	0.280
γ zone PPA (μm)	227.66±184.45	216.95±197.72	0.665	241.46±131.79	249.55±158.07	0.609
Lamina depth (μm)	473.04±159.93	450.20±164.07	0.136	437.18±159.71	483.44±162.77	**0.019**
Lamina thickness (μm)	356.84±59.63	378.15±60.78	0.101	361.81±70.12	364.06±59.27	0.825
Anterior scleral canal opening (mm)	1.97±0.48	1.95±0.25	0.763	1.85±0.17	1.88±0.19	0.193
Posterior pole characteristics						
Disc-DPE distance (mm)	2.34±1.08	2.51±1.13	0.262	2.12±0.96	2.54±1.57	**0.043**
Disc-DPE depth (μm)	58.11±49.37	71.83±150.51	0.477	67.28±51.35	110.26±141.85	**0.035**
Disc-DPE angle (deg.)	31.63±27.98	24.75±32.32	0.067	30.05±35.74	19.70±38.32	0.067
PP-CSA (mm^2^)	2.51±4.32	4.17±8.01	0.104	4.09±5.46	6.99±1.11	**0.049**
PP-HW (mm)	1.15±1.59	1.45±1.80	0.230	1.71±1.89	2.31±2.72	0.074
PP-VW (mm)	1.08±1.35	1.48±1.89	0.092	1.51±1.48	1.90±2.19	0.251

Data are presented as mean±SD unless otherwise indicated.

*Paired *t* test for continuous variables.

Statistically significant values (*P*<0.05) are shown in bold.

CSA indicates cross-sectional area; DPE, deepest point of eye; HW, horizontal width; ONH, optic nerve head; PP, posterior pole; PPA, peripapillary atrophy; VW, vertical width.

Table 3 presents the each IAD of general ocular, ONH, and posterior pole biometric measurements. Among the variables, only baseline IOP (1.38±1.31 vs. 3.44±6.79, *P*=0.019) and PP-HW (1.26±1.53 vs. 1.99±2.06, *P*=0.036) had significantly different IADs.

**TABLE 3:** Comparison of the IAD in Healthy Controls and Unilateral Glaucoma Patients

Variables	Healthy Controls	Unilateral Glaucoma	P*
Refractive error (D)	0.13±0.21	0.21±0.28	0.427
Axial length (mm)	0.38±0.76	0.21±0.24	0.210
Central corneal thickness (μm)	8.36±7.34	8.90±8.48	0.736
Baseline IOP (mm Hg)	1.38±1.31	3.44±6.79	**0.019**
Mean follow-up IOP (mm Hg)	1.15±1.03	1.68±2.29	0.105
IOP fluctuation (mm Hg)	0.35±0.77	0.63±0.84	0.075
ONH characteristics			
Horizontal tilt (deg.)	6.64±7.05	6.11±5.97	0.686
Vertical tilt (deg.)	6.31±6.14	8.11±8.29	0.198
Disc torsion (deg.)	9.31±9.97	10.26±9.69	0.626
β zone PPA (μm)	116.96±98.90	116.83±110.60	0.995
γ zone PPA (μm)	121.81±156.21	77.06±67.60	0.080
Lamina depth (μm)	90.59±82.81	96.21±90.04	0.741
Lamina thickness (μm)	54.26±39.61	53.79±38.37	0.951
Anterior scleral canal opening (mm)	0.20±0.37	0.12±0.09	0.170
Posterior pole characteristics			
Disc-DPE distance (mm)	0.92±0.81	1.02±1.22	0.626
Disc-DPE depth (μm)	26.13±53.64	28.53±46.84	0.812
PP-CSA (mm^2^)	4.13±7.14	5.95±9.52	0.259
PP-HW (mm)	1.26±1.53	1.99±2.06	**0.036**
PP-VW (mm)	1.25±1.49	1.42±1.73	0.570

Data are presented as mean±SD unless otherwise indicated. For each parameter, intereye absolute difference (IAD) was calculated by converting all left eye data to the right eye configuration and subtracting the right eye value from that of the left eye.

*Independent *t* test for continuous variables.

Statistically significant values (*P*<0.05) are shown in bold.

CSA indicates cross-sectional area; DPE, deepest point of eye; HW, horizontal width; IOP, intraocular pressure; ONH, optic nerve head; PP, posterior pole; PPA, peripapillary atrophy; VW, vertical width.

Logistic regression analysis assessing each IAD values associated with the presence of glaucoma was performed (Table 4). Univariate analysis showed that higher baseline IOP (odds ratio [OR]=1.326; 95% confidence interval [CI]=1.050-1.675; *P*=0.018), higher IOP reduction from baseline (OR=1.040; 95% CI=1.001-1.080; *P*=0.045), and larger PP-HW (OR=1.267; 95% CI=1.007-1.594; *P*=0.043) were significantly associated with the presence of glaucoma. On multivariate analysis, baseline IOP (OR=1.381; 95% CI=1.083-1.761; *P*=0.009) and PP-HW (OR=1.324; 95% CI=1.024-1.713; *P*=0.032) were the only factors significantly associated with the presence of glaucoma.

**TABLE 4:** Factors Associated With the Presence of Glaucoma in Unilateral NTG Patients With IAD Values

	Univariate		Multivariate*	
Variables	OR (95% CI)	*P*	OR (95% CI)	*P*
Axial length (mm)	0.437 (0.107-1.779)	0.248		
Central corneal thickness (μm)	1.009 (0.959-1.061)	0.734		
Baseline IOP (mm Hg)	1.326 (1.050-1.675)	**0.018**	1.381 (1.083-1.761)	**0.009**
Mean follow-up IOP (mm Hg)	1.233 (0.932-1.631)	0.142		
IOP fluctuation (mm Hg)	1.610 (0.899-2.885)	0.109		
IOP reduction from baseline (%)	1.040 (1.001-1.080)	**0.045**		
ONH characteristics				
Horizontal tilt (deg.)	0.988 (0.931-1.048)	0.683		
Vertical tilt (deg.)	1.037 (0.981-1.095)	0.199		
Disc torsion (deg.)	1.010 (0.971-1.050)	0.623		
β zone PPA (μm)	1.000 (0.996-1.004)	0.995		
γ zone PPA (μm)	0.996 (0.992-1.001)	0.093		
Lamina depth (μm)	1.001 (0.996-1.005)	0.738		
Lamina thickness (μm)	1.000 (0.990-1.010)	0.950		
Anterior scleral canal opening (mm)	0.997 (0.994-1.001)	0.117		
Posterior pole characteristics				
Disc-DPE distance (mm)	1.000 (1.000-1.000)	0.623		
Disc-DPE depth (μm)	1.001 (0.993-1.008)	0.810		
Disc-DPE angle (deg.)	1.007 (0.991-1.023)	0.413		
PP-CSA (mm^2^)	1.028 (0.979-1.079)	0.271		
PP-HW (mm)	1.267 (1.007-1.594)	**0.043**	1.324 (1.024-1.713)	**0.032**
PP-VW (mm)	1.073 (0.843-1.366)	0.568		

Data are presented as mean±SD unless otherwise indicated. For each parameter, intereye absolute difference (IAD) was calculated by converting all left eye data to the right eye configuration and subtracting the right eye value from that of the left eye.

*Variables with *P*<0.1 in the univariate analysis were included in the multivariate model.

Statistically significant values (*P*<0.05) are shown in bold.

CI indicates confidence interval; CSA, cross-sectional area; DPE, deepest point of eye; HW, horizontal width; IOP, intraocular pressure; NTG, normal tension glaucoma; ONH, optic nerve head; OR, odds ratio; PP, posterior pole; PPA, peripapillary atrophy; VW, vertical width.

**FIGURE 3:**
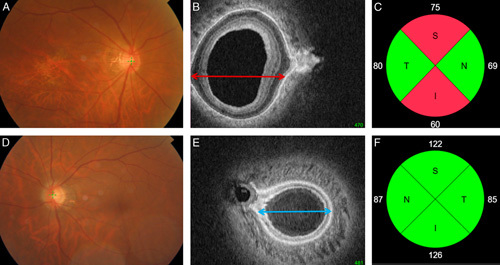
Representative case of characteristic posterior sclera shape of the glaucomatous right eye (A–C) and the healthy left eye (D–F) of a same patient. Larger posterior pole-horizontal width was a distinct feature. Red arrow designate the larger PP-HW in the glaucomatous right eye and the blue arrow designate the smaller PP-HW in the healthy left eye. I indicates inferior; N, nasal; S, superior; T, temporal.

Figure 3 represents a case of characteristic posterior sclera shape of the glaucomatous right eye (Figs. 3A–C) and the healthy left eye (Figs. 3D–F) of the same patient. Larger PP-HW was a distinct feature.

## DISCUSSION

This study introduced the use of reconstructed coronal section images to measure the Disc-DPE depth, Disc-DPE distance, PP-CSA, PP-HW, and PP-VW in human myopic eyes. These parameters were then used to estimate the size and curvature of the posterior pole, which should be considered by OCT modalities used to detect glaucomatous optic neuropathy. It also provided a conceptual framework for future OCT characterization of the posterior sclera, for glaucoma prediction and management.

Our findings suggested that glaucomatous eyes had a larger and more steeply curved posterior pole than fellow healthy eyes, based on the larger PP-CSA, PP-HW, Disc-DPE depth, and Disc-DPE distance values of the glaucomatous eyes. Furthermore, a steeply curved posterior pole was the only ocular or systemic parameter significantly associated with glaucomatous optic nerve damage. A deep posterior pole shape has been a consistent factor associated with the diagnosis and the progression of glaucomatous optic neuropathy in previous cross-sectional studies of our own.^11^,^18^

From a biomechanical perspective, IOP-related stress over extended periods of time induces remodeling and deformation of the load-bearing ONH connective tissues.^4^ In an experimental glaucoma model, ONH deformation occurred in proportion to the magnitude of IOP loading.^33^–^35^ Against this background, the results of the current study suggested that posterior segments with larger PP-CSA and PP-HW values may be subjected to significantly more remodeling than healthy eyes. Because the posterior sclera provides direct anatomic support to the ONH and its retinal ganglion cell axons, substantial remodeling of the posterior pole may result in glaucomatous optic nerve damage at the LC. A larger and more steeply curved posterior pole could be structurally disadvantageous. The total volume of the sclera remains constant even in advanced myopia.^36^,^37^ A large posterior pole would likely be composed of the relatively thin sclera, which represents a structural vulnerability.^38^ It is important to bear in mind that the posterior segment in much larger than the ONH; although the size or curvature of the posterior segment has not historically been a matter of interest for clinicians, previous results and those of this study suggest that it warrants more attention.

Measurement of the PP-HW and PP-VW provides additional information on the posterior pole. Posterior segments can show various shapes, including a perfect circle, horizontal oval, or vertical oval, even when the PP-CSA and Disc-DPE depth are the same. The curvature of the posterior pole represents a novel measurement parameter. In our study, healthy eyes had almost identical PP-HW and PP-VW values, such that the posterior segment was circular in shape. However, the difference between the PP-HW and PP-VW increased in both eyes of unilateral glaucoma patients, but especially in the glaucomatous eyes. Along with the IAD in baseline IOP, the IAD in PP-HW was the only parameter associated with glaucoma. Healthy eyes are circular, and a wide posterior segment may be because of postnatal remodeling thereof in response to stress and strain in the horizontal direction. PPA may represent additional structural evidence of horizontal postnatal remodeling. Temporal enlargement of the area of PPA in children has been documented.^39^ Kim et al^19^ suggested that PPA development over time may be because of scleral stretching. The main cause of scleral stretching may be the horizontal widening of the posterior sclera. Substantial stress and strain in the horizontal direction can induce a wide posterior pole and enlargement of the PPA. This hypothesis is supported by computer simulations, which suggested that horizontal eye movements cause a high degree of strain, especially where the dura and sclera bind in the horizontal direction.^40^–^42^ Repeated horizontal pulling force at this location would pull the posterior sclera in a horizontal direction, eventually resulting in a PP-HW that is larger than the PP-VW.

In this study, there were no intereye differences in any ONH parameter, unlike the Disc-DPE distance, Disc-DPE depth, and PP-CSA. However, previous studies reported that ONH parameters were associated with the presence of glaucoma.^43^–^45^ This discrepancy may be because of differences in participant characteristics among studies, where myopic patients are more likely to have glaucoma with increasing size of the PPA area or degree of optic disc tilt.^20^,^46^ Such studies have provided clear evidence of ONH changes in myopic patients.^44^,^47^ Our study group had excluded highly myopic subjects with <−6.0 D, which for some patients might not develop evident ONH changes. However, this also reinforces our claim that the posterior sclera changes could accomplish the detection of glaucomatous changes in another aspect.

The ONH is surrounded by the peripapillary sclera, which has a collagen layer and abundant elastin.^48^ This organization of the peripapillary sclera provides a buffer to absorb the stress to which the posterior segment is subjected, and attenuates morphologic changes in the ONH in nonmyopic subjects, but exaggerated posterior segments of myopic subjects may not have sufficient buffer activity to reduce or prevent ONH deformation. In addition, our results clearly show that the difference in the posterior pole anatomy is in far greater scale than the ONH, even in the nonmyopic glaucoma subjects. Thus, we carefully suggest that examining the posterior sclera biometry may lead to early detection of glaucoma.

We suggest using the size and curvature of the posterior segment as markers for glaucoma, similar to keratoconus, which can be diagnosed and classified according to the degree of curvature, and morphology and thickness of the cone.^49^,^50^ The staging of keratoconus is based on the prognosis and visual acuity, whereas glaucoma is staged on the basis of the degree and etiology of IOP, which has a weak correlation with the disease prognosis.^51^ Although there have been extensive efforts to characterize the pathophysiology of glaucoma, the clinical classification does not seem to be based on the prognosis of the disease, or the changes in visual acuity that it causes. Increasing use of corneal topography has rendered the disease course of keratoconus more predictable.^52^ Our results suggest that glaucoma could be approached in a similar manner, although not as a replacement for the existing approach on the basis of IOP measurement. Our data clearly showed that the change in baseline IOP is the most useful parameter associated with glaucoma. Elevated IOP is an important risk factor for glaucoma, and is the principal modifiable factor in the treatment thereof.^53^–^56^

This study had several limitations, including the use of a cross-sectional design preventing the determination of causality in the relationship between glaucomatous axonal damage and posterior pole size or curvature. In addition, the remodeling of the posterior pole can only be hypothesized and cannot be assumed. Second, all of the participants were Korean, where the effects of the posterior sclera structure on glaucoma might be different in other populations. Third, counting the number of coronal sections to estimate tissue depth does not yield a precise measurement, but rather only an estimate. However, there is still no other way to measure the depth of the posterior sclera on a real-time scan, and our methodology was adequate for comparing the DPE among participants. Fourthly, defining a definite landmark of the posterior LC is abstruse. However, there still is a vague difference at the posterior border and the optic nerve parenchyma. In this study, 2 experienced ophthalmologists (Y.C.K. and K.D.K.) who were masked to the subjects’ clinical information performed the measurements and the disagreements were resolved by consensus between the 2 ophthalmologists or a third adjudicator (H.B.H.). Finally, the DPE was stable only when the participants’ eyes were fixated on the scanning light. However, all ocular imaging instruments assume that the subject is fixating on the scanning light so, like any other ocular parameter, such as PPA, optic disc tilt, and optic disc torsion, the DPE is reproducible as long as the proper fixation is achieved.

In summary, our study quantified the size and curvature of the posterior pole in unilateral glaucomatous eyes, and determined the associations of these parameters with glaucoma. Such parameters may be useful for predicting the susceptibility of eyes to glaucomatous damage, and could form the basis of new and more accurate diagnostic tests.

## Supplementary Material

SUPPLEMENTARY MATERIAL

Supplemental Digital Content is available for this article. Direct URL citations appear in the printed text and are provided in the HTML and PDF versions of this article on the journal's website, www.glaucomajournal.com.
