# Supplementary material for: The Shape of Posterior Sclera as a Biometric Signature in Open-angle Glaucoma: An Intereye Comparison Study
Source: J Glaucoma. 2020 Jun 16;29(10):890–8. doi: 10.1097/IJG.0000000000001573 (PMC7647446; doi:10.1097/IJG.0000000000001573)
Supplement: SUPPLEMENTARY MATERIAL [file ijg-29-890-s001.docx]

**Supplementary Table 1.** Reproducibility of the measurements using the swept-source optical coherence tomography inbuilt caliper.

|  | **Intraobserver ICC**† |  | **Interobserver ICC**† |
| --- | --- | --- | --- |
| Disc-DPE distance | 0.972 (0.880–0.991) |  | 0.936 (0.910–0.969) |
| Disc-DPE depth | 0.998 (0.995–0.999) |  | 0.998 (0.995–0.999) |
| Disc–DPE angle | 0.965 (0.955–0.990) |  | 0.964 (0.904–0.984) |
| PP-CSA | 0.865 (0.815-0.980) |  | 0.821 (0.805-0.924) |
| PP-HW | 0.990 (0.982-0.999) |  | 0.996 (0.994-0.999) |
| PP-VW | 0.990 (0.960-0.999) |  | 0.982 (0.963-0.995) |
| Beta zone PPA | 0.935 (0.910-0.972) |  | 0.911 (0.902-0.966) |
| Gamma zone PPA | 0.945 (0.920-0.967) |  | 0.957 (0.910-0.978) |
| Lamina depth | 0.891 (0.822-0.933) |  | 0.841 (0.792-0.913) |
| Lamina thickness | 0.878 (0.842-0.913) |  | 0.779 (0.731-0.892) |

CSA: cross sectional area; DPE: deepest point of the eye; ICC: intraclass correlation coefficient; HW: horizontal width; ONH: optic nerve head; PP: posterior pole; PPA: peripapillary atrophy; VW: vertical width

*ICC for single measure.

†ICC for average measure.
